# Orange Leafhopper *Cicadulina bipunctata* Feeding Induces Gall Formation Nitrogen Dependently and Regulates Gibberellin Signaling

**DOI:** 10.3390/plants9101270

**Published:** 2020-09-26

**Authors:** Sho Miyazaki, Keita Kasahara, Soh Matsui, Makoto Tokuda, Yoko Saikawa

**Affiliations:** 1 Faculty of Science and Technology, Keio University, 3-14-1 Hiyoshi, Kohoku-ku, Yokohama 223-8522, Japan; keitakasahara@keio.jp (K.K.); atamatsuisoh@keio.jp (S.M.); 2 Faculty of Agriculture, Saga University, 1 Honjo-machi, Saga 840-8502, Japan; tokudam@cc.saga-u.ac.jp

**Keywords:** insect feeding, gall formation, growth suppression, plant hormones, nutrient levels

## Abstract

Orange leafhopper *Cicadulina bipunctata* feeding induces wallaby ear symptoms, namely growth suppression and gall formation characterized by severe swelling of leaf veins, on various Poaceae, thereby leading to low crop yields. Here, we investigated the development of wallaby ear symptoms on rice seedlings due to *C. bipunctata* feeding. After confirming that *C. bipunctata* feeding induces growth suppression and gall formation on rice seedlings, we further demonstrated that gall formation score decreased with decreasing levels of nitrogen in the medium and that *C. bipunctata* feeding induces the expression levels of nitrogen transporter genes. These gene expression changes may participate in the nutrient accumulation observed in galled tissues and in gall formation. In addition, these expression changes should induce growth promotion but the inhibition of gibberellin signaling by *C. bipunctata* feeding might be the reason why growth is suppressed. Treatment with plant growth regulators did not affect gall formation, suggesting the existence of a complex gall formation mechanism by *C. bipunctata* feeding.

## 1. Introduction

**Figure 1:**
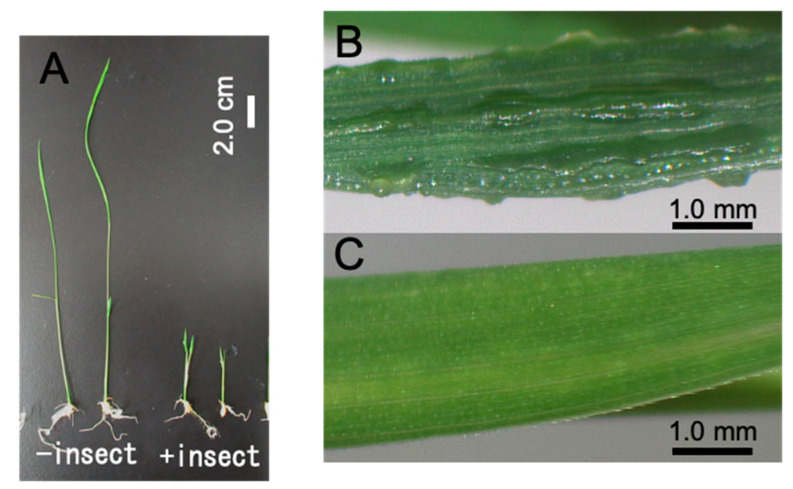
Wallaby ear symptoms on rice seedlings. Wallaby ear symptoms generated on rice seedlings grown on peat moss medium by the feeding of *C. bipunctata.* (**A**) Growth suppression of rice seedlings, (**B**) gall formation and severe swelling of leaf veins, and (**C**) control leaf with no *C. bipunctata* feeding.

The orange leafhopper *Cicadulina bipunctata* (Melichar) is distributed widely across eastern and northern Africa, the Indian Ocean, the southern Palearctic, eastern Japan, namely Shikoku, Kyushu, and the Bonin Islands, and southern to northern Australia [1,2,3]. This species is considered a serious pest of maize, rice, and wheat because *C. bipunctata* feeding induces growth suppression and severe swelling of leaf veins (Figure 1), both of which leading to the loss of leaf and stem dry matter in maize and resulting in yield loss [4]. However, yield reductions caused by *C. bipunctata* feeding are low in tolerant maize at the field stage, and both growth suppression and severe swelling of leaf veins can be induced on young maize but not on maize at the fifth leaf stage or older [4]. These symptoms were previously assumed to be caused by a leafhopper-transmitted virus [5,6]. However, Ofori and Francki showed that the symptoms could be induced in maize by virus-free *C. bipunctata* [7], and no viruses were observed by transmission electron microscopy in the swelled leaf tissues of maize [8]. Matsumura and Tokuda established a simple method for evaluating these symptoms using the varietal resistance of maize and rice, which partially revealed the generation process of wallaby ear symptoms in maize [9,10]. Specifically, when *C. bipunctata* adults were released on a young maize seedling, growth suppression and swelling of leaf veins were evident on a new leaf extending from the seedling but not on the *C. bipunctata* feeding site. In addition, the symptoms did not appear when further new leaves were produced after the removal of *C. bipunctata* [11]. Therefore, the swelling of leaf veins is regarded not as a viral disease but as an insect gall caused by the chemical substance(s) secreted by insects; thus, it is now termed a “wallaby ear symptom” not “wallaby ear disease.”

Galls are structures produced by plants in response to the activity of several types of organisms, including insects. Galling insects have a close relationship with their host plants, as their habitat is largely restricted to the gall and plant organ in which the gall is developed. Three hypotheses have been proposed for the adaptive significance of gall induction: the enemy hypothesis, the nutrition hypothesis, and the microenvironment hypothesis [12]. In general, galling insects induce galls at their feeding sites and increase their own fitness. In the case of *C. bipunctata*, gall induction does not seem to contribute to improve their own performance because feeding and gall formation sites are different, and adults of *C. bipunctata* are able to move to other host plants in the field. Nevertheless, the amino acid levels and glucose content of galled leaves are increased by *C. bipunctata* feeding, and the emergence and developmental rate of nymphs also increase when they feed on galled leaves [13]. This suggests that gall tissues contribute to the improved performance of *C. bipunctata* offspring. The involvement of plant hormones in gall formation has long been suggested due to their presence in galling insects [14,15,16,17,18,19]. Cytokinins including *trans*-Zeatin (tZ) centrally regulate cell division and differentiation in plants and gibberellins (GAs) promote seedling elongation. In galled maize leaves, higher content of cytokinin and lower content of GAs were found than in ungalled leaves [20]. Such hormonal changes have not been observed when *C. bipunctata* was released in the leaves of wallaby ear symptom-resistant maize, indicating that plant hormones contribute to gall formation.

The present study aimed to investigate the physiological activity of wallaby ear symptom on rice due to *C. bipunctata*. We first examined the relationships between nutrients of plant growth medium and wallaby ear symptom, growth suppression, and gall formation. Secondly, we investigated the effect of plant hormones and its regulators on growth suppression and gall formation in rice.

## 2. Results

### 2.1. Wallaby Ear Symptoms on Rice Seedlings

Because nymphs hatched during the assay, *C. bipunctata* females were not suitable for the bioassay; hence, only males were used (Figure S1). We first confirmed whether the wallaby ear symptoms induced in rice seedlings (Nipponbare variety) were similar to that of maize seedlings [9,21]. Agar, half-strength Murashige and Skoog (MS), MS, and peat moss were used as media for the bioassay. Figure 1 shows the induction of wallaby ear symptoms, growth suppression (Figure 1A) and gall formation (Figure 1B,C) in rice seedlings using peat moss as the medium.

**Figure 2:**
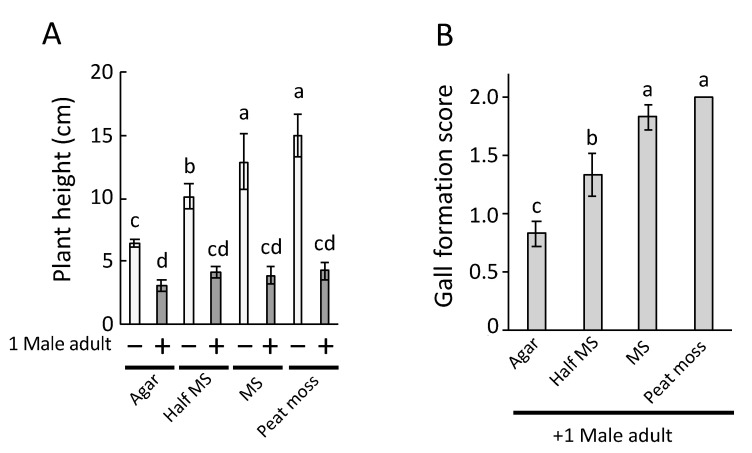
Effects of media on wallaby ear symptoms. Rice seedlings were transferred onto new agar, half-strength Murashige and Skoog (MS) (half MS), MS, or peat moss media with/without one *C. bipunctata* male. (**A**) Seedling length measured on day 4. Comparisons are based on two-way ANOVA (Table S2) followed by Tukey’s honestly significant difference (HSD) test. Bars with different letters indicate significant differences (*p* < 0.05). Error bars indicate the standard deviation, *n* = 12. (**B**) Gall formation score recorded on day 10. Bars with different letters indicate significant differences (*p* < 0.05) using Tukey’s HSD test. Error bars indicate the standard deviation, *n* = 12.

Figure 2 shows the results of the whole bioassay. Seedling growth was significantly suppressed by the feeding of *C. bipunctata* in all tested media (Figure 2A, *p* < 0.05, Tukey’s honestly significant difference (HSD) test). The two-way analysis of variance (ANOVA) revealed that both feeding of *C. bipunctata* and media had a statistically significant effect on seedling length, and there was an interactive effect between feeding of *C. bipunctata* and media (Table S2). After 10 days of cultivation, we evaluated the score of gall formation. Nutrients levels differed across media, and Tukey’s HSD test demonstrated that agar, half-strength MS, and MS significantly differed from each other (*p* < 0.05) but no significant difference was observed between MS and peat moss (*p* = 0.77). In contrast to seedling length, with increasing levels of nutrients (1.0% agar < half-strength MS < MS, peat moss), gall formation by the feeding of *C. bipunctata* also substantially increased on the rice seedlings (Figure 2B).

### 2.2. Nitrogen Requirement for Gall Formation on Rice Seedlings

**Figure 3:**
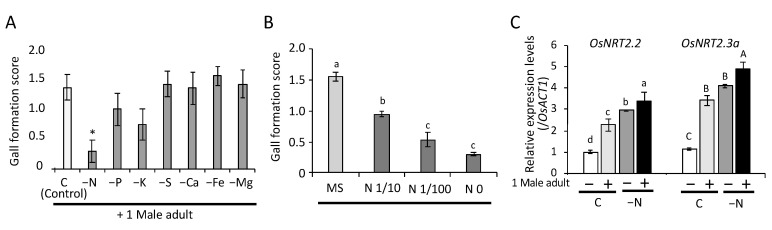
Effects of nutrient deficiency on gall formation. (**A**) Rice seedlings were transferred onto new MS media without N, P, K, S, Ca, Mg, or Fe, and male *C. bipunctata* were added. C—control. Gall formation score was recorded 10 days after samples were transferred to each type of media. Dunnett’s test (*, *p* < 0.01 versus control) was performed to evaluate differences among treatments. Error bars indicate the standard deviation, *n* = 16 (-Ca and -Fe) and *n* = 18 (C, -N, -P, -K -S and -Mg). (**B**) Gall formation score decreased as N decreased. Bars with different letters indicate significant differences (*p* < 0.05) with Tukey’s HSD test. Error bars indicate the standard deviation, *n* = 20 (MS and N 1/10) and *n* = 23 (-N and N 1/100). (**C**) *C. bipunctata* feeding induced the expression of rice N-acquisition gene *OsNRT2.2* and NO_3_^−^ long distance transporter gene *OsNRT2.3a*. C; control (MS media). Bars with different letters indicate significant differences (*p* < 0.05) with Tukey’s HSD test. Error bars indicate the standard deviation, *n* = 3.

Due to the significant difference in the generation of galls between MS and agar media, we further investigated whether gall formation depended on nutrient levels. We cultivated rice seedlings in MS media without nitrogen (N), phosphorous (P), potassium (K), sulfur (S), calcium (Ca), magnesium (Mg), or iron (Fe) with or without *C. bipunctata*. Gall generation only significantly decreased in the absence of N (*p* < 0.001, Dunnett’s test) (Figure 3A). Thus, the correlation between N and gall formation was further evaluated. When the N source (NH_4_NO_3_ and KNO_3_) was reduced to 1/10 and 1/100 of that in the control MS medium, the gall formation score significantly decreased as N decreased; however, no further reduction was observed between 1/100 and zero N (*p* = 0.35, Tukey’s HSD test) (Figure 3B). In contrast, growth suppression by the feeding of *C. bipunctata* was not affected by nutrient levels (Figure S3). The two-way ANOVA revealed that feeding of *C. bipunctata* significantly suppressed growth in all tested conditions, but there was no interactive effect between feeding and nutrient deficiency (Table S2). Lack of N, K, or Fe led to growth inhibition compared to control seedlings under *C. bipunctata* non-feeding conditions, but no significant difference was observed among *C. bipunctata* feeding conditions. Considering all single macronutrient-deficient media, significant differences were observed between *C. bipunctata* feeding and non-feeding conditions (Figure S3).

Since N is required for gall formation, we investigated the effect of *C. bipunctata* feeding on N-uptake- and transport-related gene expression. Nitrogen is an essential nutrient for plant growth and development and for the uptake of nitrate ions (NO_3_^−^), which is mediated by nitrate transporters (NRTs). In rice, NO_3_^−^ can be directly transported to the xylem from the root by the OsNRT2.3a transporter [22]. To determine whether N-uptake- and transport-related genes responded to *C. bipunctata* feeding, we examined gene expression levels of rice seedlings cultivated on MS or N-deficient media with or without *C. bipunctata* for 4 days. As shown in Figure 3C, the expression levels of the rice N-acquisition gene *OsNRT2.2* and NO_3_^−^ long distance transporter gene *OsNRT2.3a* in the root were significantly increased in N-deficiency treatments (*p* < 0.001, Tukey’s HSD test), as reported previously [23]. Moreover, *C. bipunctata* feeding induced the expression of both genes even under N-deficient conditions (*p* < 0.05, Tukey’s HSD test).

### 2.3. Regulation of GA Signaling by C. bipunctata Feeding

**Figure 4:**
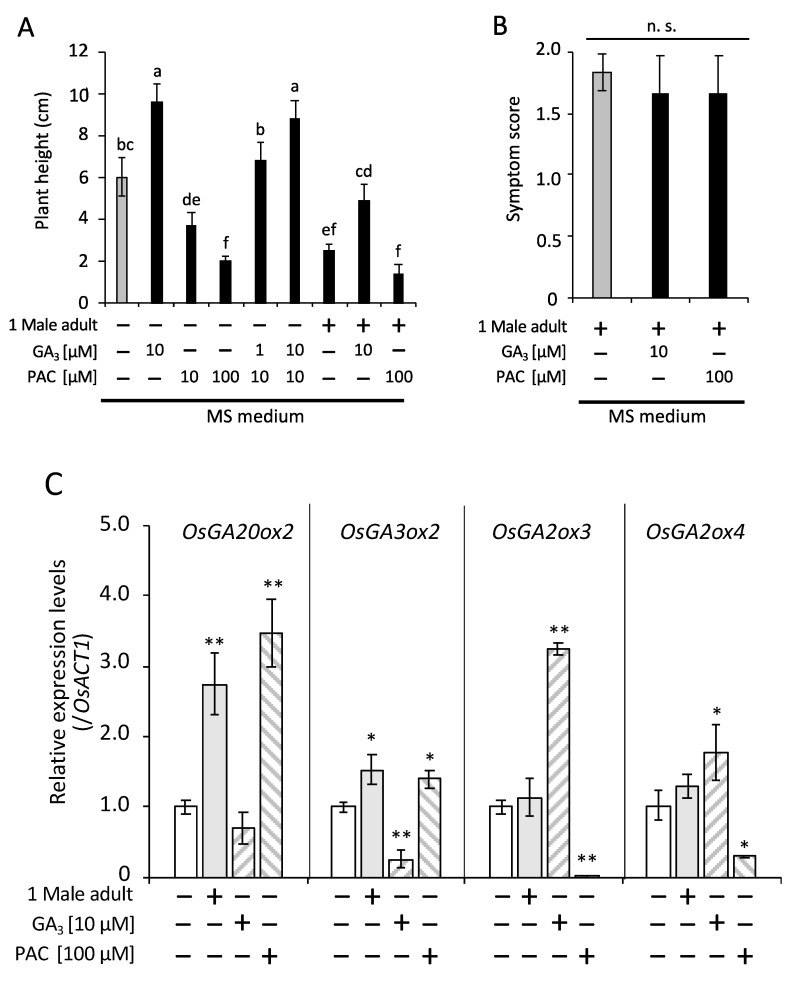
Effects of gibberellin (GA) signaling on wallaby ear symptoms. The rice seedlings were placed on MS containing GA_3_ or/and paclobutrazol (PAC) or 0.1% dimethyl sulfoxide (DMSO) as a mock control with or without a male *C. bipunctata*. (**A**) The length of seedlings was measured on day 4 and comparisons were made by two-way ANOVA followed by Tukey’s HSD test. Bars with different letters indicate significant differences (*p* < 0.05). Error bars indicate the standard deviation, *n* = 6. (**B**) The gall formation score was recorded 10 days after samples were transferred to each type of media. There was no significant interaction between insect feeding and PAC/GA treatment (one-way ANOVA, *F* (2, 15) = 3.68, *n* = 10, *p* = 0.90). Error bars indicate the standard deviation, n. s.; not significant. (**C**) Transcript levels of GA-biosynthetic genes (*OsGA20ox2* and *OsGA3ox2*) and GA-catabolic genes (*OsGA2ox3* and *OsGA2ox4*) are relative to those of *OsACT1* (used as the internal control). Dunnett’s test (* *p* < 0.05, ** *p* < 0.01 versus (-)). Error bars indicate the standard deviation, *n* = 3.

It has been reported that *C. bipunctata* feeding significantly decreased GA contents and increased cytokinin contents in extending galled leaves of maize [20]. We thus investigated whether wallaby ear symptoms in rice seedlings was regulated by GA and/or its biosynthetic inhibitor paclobutrazol (PAC). The two-way ANOVA revealed that both feeding of *C. bipunctata* and PAC/GA treatment had a statistically significant effect on seedling length, and there was an interactive effect between feeding of *C. bipunctata* and PAC/GA treatment (Table S2). As shown in Figure 4A, growth suppression by 10 µM PAC was recovered by the application of 1 µM GA (*p* < 0.001, Tukey’s HSD test) in the non-feeding condition. In the *C. bipunctata* feeding condition, growth was suppressed as in the 10 µM PAC treatment, but it did not recover sufficiently after the application of 10 µM GA. However, the growth suppression by *C. bipunctata* feeding was further suppressed by the application of 100 µM PAC, similar to that of seedlings with 100 µM PAC under *C. bipunctata* non-feeding conditions (*p* = 0.18, Tukey’s HSD test). No significant difference was observed in gall formation occurrence regardless of the supplementation of GA or PAC (Figure 4B, one-way ANOVA, *F* (2, 15) = 3.68, *p* = 0.90). These data showed that *C. bipunctata* feeding causes rice plants to be less responsive to GA. Therefore, we investigated the effect of *C. bipunctata* feeding on the expression of GA-biosynthetic and -catabolic rice genes. As shown in Figure 4C, the transcript levels of the GA-biosynthetic genes *OsGA20ox2* and *OsGA3ox2* were significantly increased by the PAC treatment and decreased by the GA treatment, with the exception of *OsGA3ox2*. The transcript levels of GA-catabolic genes *OsGA2ox3* and *OsGA2ox4* were significantly decreased by the PAC treatment and increased by GA treatment (*p* < 0.05, Tukey’s HSD test). In the *C. bipunctata* feeding condition, the transcript levels of the GA-biosynthetic genes were significantly increased but no significant difference was observed in GA-catabolic genes. Thus, *C. bipunctata* feeding not only reduced GA responsiveness, but also induced the expression of the GA biosynthetic genes.

Next, the influence of cytokinin on wallaby ear symptoms was examined. It has been demonstrated that growth inhibition of rice seedlings by cytokinin is suppressed by the chemical regulator S-4893 through its antagonistic action on cytokinin receptors [24]. Therefore, we used this compound for the bioassay. We found that neither cytokinin tZ or S-4893 had a significant effect on growth or gall formation with *C. bipunctata* feeding (Figure S4). In addition to GA and tZ, we also investigated the effect of other plant hormones on wallaby ear symptom; however, neither brassinosteroid (BL), its inhibitor brassinazol (Brz), auxin (IAA), or its receptor antagonist α-(phenylethyl-2-oxo)-IAA (PEO-IAA) had significant effects on growth suppression and gall formation with *C. bipunctata* feeding (Figures S5 and S6).

## 3. Discussion

It has been reported that *C. bipunctata* feeding induces wallaby ear symptoms, namely growth suppression and gall formation characterized by severe swelling of leaf veins, on various Poaceae including the major staple crops maize, rice, and wheat [21]. In agreement with previous studies, the present results showed that *C. bipunctata* induces both symptoms in rice seedlings [9,21]. In all tested media, gall formation score tended to increase as nutrient levels also increased, and a correlation was found between gall formation and N content. This result suggested that N was required for gall formation.

We also demonstrated that the expressions of genes related to N-uptake and N-transport were increased by *C. bipunctata* feeding, even in N-deficiency conditions. It has been reported that the expressions of *OsNRT2.1* and *OsNRT2.3a* respond not only to nitrate concentration but also to drought stress [25]. Yang et al. reported that rice infected by the endophytic fungus *Phomopsis liquidambari* showed substantial changes in N uptake and N metabolism [26]. The transcript levels of N-assimilation genes including *OsNRT2.1* were higher in infected than in uninfected tissues, and the transcript levels in endophyte-infected tissue was highest under low N conditions. Thus, Yang et al. indicated that low N-fertilization induces a physiological state of rice that is favorable to *P. liquidambari* symbiosis, and that the resulting higher infection by the fungal endophyte is necessary for the positive effects observed in rice performance [26]. *C. bipunctata* also increases the amino acid and glucose levels in the galled leaves of maize thereby suggesting that a N source is required for N-rich galls [13]. Taken together, the results of previous studies and that or the present study suggest that N-uptake associated with gene expression changes may participate in the nutrient accumulation observed in galled tissues, in gall formation, and in nutrient-rich food for *C. bipunctata* feeding. It is not clear how N is transported to the gall and why *C. bipunctata* suppress the growth of the plant they feed upon.

The mechanism of gall formation has been studied for a long time, but it is still unclear. Some insects induce galls in areas in which they do not feed, similarly to *C. bipunctata* [27]. In addition, the application of extracts prepared from gall-inducing insects to host plants induces morphological changes [28,29,30]. These findings suggest that bioactive chemical substances injected by the insects move within host tissues and influence gall formation. It has been proposed that plant hormones play important roles in gall formation and maintenance. In fact, galled maize leaves due to *C. bipunctata* feeding contain higher contents of tZ and lower contents of GAs than ungalled leaves [20]. Hence, we performed a bioassay using tZ, GA, and their signal/biosynthesis inhibitors to determine whether wallaby ear symptoms are affected by these plant hormones. Notably, such treatments did not affect gall formation. Thus, information on how plant hormones are involved in gall formation is still needed to help elucidate the gall formation mechanism. The other wallaby ear symptom, growth inhibition, was affected by both GA and its inhibitor PAC. Growth suppression by *C. bipunctata* feeding was similar to that of rice seedlings treated with 10 µM PAC. The rice seedlings growth suppressed by treatment with 10 μM PAC was recovered by applying 1 µM GA and accelerated by 10 µM GA, whereas the growth suppression by *C. bipunctata* feeding did not completely compensated by applying 10 µM GA. Moreover, *C. bipunctata* feeding was not significantly accelerated by the application of 100 µM PAC. In addition, *C. bipunctata* feeding induced the expression of the two GA-biosynthetic genes tested but not that of the two GA-catabolic genes tested. Consistent with previous studies in maize [20], this gene expression pattern implies that GA content is low in plants. These results implied that GA signaling and/or GA-biosynthesis, but not GA-catabolism, was likely impaired by *C. bipunctata* feeding, resulting in suppressed rice growth. Recently, Yasui et al. showed that downregulation of the transcription factor *OsWOX4*, which regulates shoot apical meristem maintenance, induces severe defects in leaf development [31]. The chemical substance(s) injected by *C. bipunctata* during feeding may directly or indirectly affect the shoot apical meristem, which harbors a group of stem cells that induce growth suppression. This hypothesis needs further exploration; therefore, future research should examine the transcriptional regulation of GAs modulating the expression of *OsWOX*.

A previous study showed that barley does not exhibit any wallaby ear symptoms, unlike other Poaceae, and that the two symptoms could be independently suppressed by using six barley chromosome disomic addition lines [32]. However, it is still unclear whether both phenomena are regulated by a single chemical substance. That is, one or more chemical substances injected by *C. bipunctata* and inducing gall formation and growth inhibition may regulate the expressions of genes related to N-uptake and transport and may inhibit GA signaling to suppress rice growth. Further studies are needed to elucidate the mechanism of wallaby ear symptoms.

## 4. Materials and Methods

### 4.1. Materials and Growth Conditions

*Cicadulina bipunctata* were obtained from Kyushu Okinawa Agricultural Research Center, NARO, and wild rice (Nipponbare variety; Nouken Co., Ltd., Misato, Japan) was used in all experiments (Figure S1). *C. bipunctata* egg-laid rice seedlings were incubated in an insect-proof cage (Asahi, Tokyo, Japan) at 28.5 °C under an 8 h/16 h dark/light cycle; nymphs were hatched after one-week incubation. For the rice seedling tray exchange, rice seeds were sterilized with sodium hypochlorite and germinated in water for 48 h at 28.5 °C. Germinated seeds were sown on peat moss, the fibrous product of sphagnum moss and other organic materials that decompose in peat bogs (Hokkaido Peat Moss, Saitama, Japan), in the plastic tray of the insect-proof cage and cultivated for 4 days. When renewing the rice seedling tray, the old rearing container was turned up and the new rearing container was turned upside down, which caused the insects inside to drop onto the new rice seedling tray. Rice seedling tray exchange was conducted weekly. Emerged adults were obtained after four weeks, and then laid eggs.

### 4.2. Bioassay

#### 4.2.1. Preparation of Rice Seedlings

Rice seeds were sterilized for 10 min in 10% sodium hypochlorite and germinated in water for 48 h at 28.5 °C. The germinated seeds were planted in 1.0% (*w*/*v*) agar and cultivated for 48 h at 28.5 °C under an 8 h/16 h dark/light cycle. Germinated seedlings were used in all experiments.

#### 4.2.2. Effects of Plant Growth and Gall Induction by *C. bipunctata* on Rice Seedlings

To examine the generation of wallaby ear symptoms by *C. bipunctata*, 1.0% agar, half-strength MS medium (Nihon Seiyaku, Tokyo, Japan) with 1.0% (*w*/*v*) agar, MS medium with 1.0% (*w*/*v*) agar, and peat moss were used in the tests. Each germinated seedling was transferred onto 1.0% (*w*/*v*) agar, half MS, MS, or peat moss contained in 1.5-mL tubes. Then, each tube plus one *C. bipunctata* male was placed into a glass tube (17.5 × 135 mm) with a lid containing a hole and cultivated at 28.5 °C under an 8 h/16 h dark/light cycle. A tube containing no insect was used as the control for each medium. After 4 days of cultivation, the length of seedlings was measured using ImageJ software (National Institutes of Health, Bethesda, MA, USA). After 10 days of cultivation, formed galls were observed using a microscope (Shimadzu STZ-171, Shimadzu Corp., Kyoto, Japan) and the symptom scores (2 = tissues heavily swollen; 1 = veins partially thickened; 0 = no visible symptoms) established by Matsumura and Tokuda were recorded (Figure S2) [9], *n* = 12. These experiments were repeated three times with similar results.

#### 4.2.3. Effects of Nutrient Deficiency on Plant Growth and Gall Induction

To evaluate the effects of nutrient levels on plant growth and gall induction, a germinated seedling was planted in the nutrient-sufficient medium (control) or in a single macronutrient deficient medium (i.e., without N, P, K, S, Ca, Mg, or Fe) with 1.0% (*w*/*v*) agar in 1.5-mL tubes (Table S1). Then, the tubes and one *C. bipunctata* male per tube were placed into glass tubes (17.5 × 135 mm) with a lid containing a hole and cultivated at 28.5 °C under an 8 h/16 h dark/light cycle. A tube containing no insect was used as the control. All media contained 1 mM 2-(*N*-morpholino) ethanesulfonic acid buffer (pH 5.7) [33]. Four days later, the length of seedlings was measured, *n* = 5. Symptom scores were recorded after 10 days of cultivation, as described above, *n* = 16 (-Ca and -Fe) and *n* = 18 (C, -N, -P, -K -S and -Mg). These experiments were repeated three times with similar results.

#### 4.2.4. Effects of Plant Hormones on Plant Growth and Gall Induction

To examine the effect of plant hormones on plant growth and gall induction, seedlings were planted in MS agar medium with 1.0% (*w*/*v*) plant hormones and its regulators, i.e., GA_3_ (FUJIFILM Wako Pure Chemical Corporation, Osaka, Japan), PAC (FUJIFILM Wako Pure Chemical Corporation, Tokyo, Japan), cytokinin-signaling inhibitor S-4893 (Enamine Ltd., Ukraine) [24], cytokinin tZ (FUJIFILM Wako Pure Chemical Corporation), auxin (IAA, FUJIFILM Wako Pure Chemical Corporation), IAA receptor inhibitor (PEO-IAA, Med Chem Express, NJ, USA) [34], brassinolide (BL, Cayman Chemical Company, MI, USA), BL biosynthesis inhibitor brassinazol (Brz, Cayman Chemical Company), or dimethyl sulfoxide (DMSO) as a mock control, and cultivated at 28.5 °C under an 8 h/16 h dark/light cycle with or without one male adult of *C. bipunctata*. Four days later, the length of seedlings was measured, *n* = 6 (PAC/GA, tZ/S4893 and IAA/PEO-IAA) and *n* = 5 (BL/Brz). Symptom scores were recorded after 10 days of cultivation, as described above, *n* = 10 (PAC/GA) and *n* = 5 (tZ/S4893, IAA/PEO-IAA and BL/Brz). These experiments were repeated three times with similar results.

#### 4.2.5. Preparation of the Samples for Quantitative Real-Time PCR

To examine the effects of *C. bipunctata* feeding on the expressions of genes related to N-uptake and transport, a germinated seedling was transferred onto MS medium or N-deficient MS medium in 1.5-mL tubes. Then, the tubes and one *C. bipunctata* male per tube were placed into glass tubes (17.5 × 135 mm) with a lid containing a hole and cultivated for 4 days at 28.5 °C under continuous light. A tube containing no insect was used as the control. To examine the effects of *C. bipunctata* feeding on GA-related gene expression, a germinated seedling was transferred onto MS medium containing GA_3_, PAC, or 0.1% DMSO as mock control in 1.5-mL tubes. Then, the tubes and one *C. bipunctata* male per tube were placed into glass tubes (17.5 × 135 mm) with a lid containing a hole and cultivated for 4 days at 28.5 °C under an 8 h/16 h dark/light cycle. A tube containing no insect was used as the control.

### 4.3. Quantitative Real-Time PCR Analysis

Total RNA was extracted from frozen plant material using PureLink Plant RNA Reagent (Invitrogen, Life Technologies, CA, USA) according to the manufacturer’s instructions. Five hundred nanograms of each RNA sample was used for cDNA synthesis with the ReverTra Ace^®^ qPCR RT Master Mix (TOYOBO, Tokyo, Japan). Quantitative real-time PCR was performed as described previously [35]. Three identically treated replicates of each RNA sample were analyzed to account for biological variation, *n* = 3. The primers for genes *OsACT1* (encoding *actin1*), *OsGA20ox2* (encoding GA20-oxidase 2, LOC_Os01g66100), *OsNRT2.2* (LOC_Os02g02190), and *OsNRT2.3a* (LOC_Os01g50820) were used as described previously [36,37,38]. *OsGA2ox3* (encoding GA2-oxidase 3, LOC_Os01g55240), *OsGA2ox4* (encoding GA2-oxidase 4, LOC_Os05g43880), and *OsGA3ox2* (encoding GA -oxidase 2, LOC_Os01g08220) were amplified with the following primers: *OsGA2ox3-*forward (5′-TTCTTCGTCAACGTCGGCGACTCGTTGC-3′) and -reverse (5′-TCTCAAACTGGGCCAGCCTGTTGTCTCC-3′); *OsGA2ox4-*forward (5′-GCGTGCGAGAGGTTTGGGTTCTTCAAGG-3′) and -reverse (5′-CTCCGCCACCATCTCCAGCACCGTCC-3′); *OsGA3ox2-*forward (5′-TCCTCCTTCTTCTCCAAGCTCAT -3′) and -reverse (5′-GAAACTCCTCCATCACGTCACA-3′). The quantitative real-time PCR experiment was repeated two times with similar results.

### 4.4. Statistical Analyses

All data are shown as means ± standard deviations. Statistical analyses, Tukey’s HSD test, and Dunnett’s test were performed using the R software (https://www.r-project.org/).

#### 4.4.1. Plant Growth

To examine the effects of *C. bipunctata* feeding on plant growth, a two-way ANOVA followed by Tukey’s HSD test were used to evaluate significant differences among *C. bipunctata* feeding and plant growth media or GA/PAC treatments. For all tests, *p* < 0.05. To evaluate *C. bipunctata* feeding effects on plant growth, a two-way ANOVA followed by Dunnett’s test was applied to examine differences between *C. bipunctata* feeding and single-nutrient deficiency. For all tests, significance was assessed at levels of *p* < 0.05 and *p* < 0.01. A two-sided Student’s *t*-test was conducted to determine significant differences in seedling length between *C. bipunctata* feeding and non-feeding treatments.

#### 4.4.2. Gall Formation

To examine the effect of *C. bipunctata* feeding on gall formation score, a Tukey’s HSD test was used to evaluate significant differences between *C. bipunctata* feeding and plant growth media treatments. For all tests, *p* < 0.05. The Dunnett’s test was applied to examine differences between *C. bipunctata* feeding and nutrient deficiency. For all tests, *p* < 0.01. To examine *C. bipunctata* feeding effects on the expression levels of N-uptake and transport genes, we used Tukey’s HSD multiple comparison test. To examine the *C. bipunctata* feeding effects on the expression levels of GA-biosynthesis and -catabolic genes, we used Dunnett’s multiple comparison test. In these tests, *p* < 0.05 and *p* < 0.01. To assess the effects of plant growth regulators on gall formation, a one-way ANOVA was performed.

## Supplementary Materials

The following are available online at https://www.mdpi.com/2223-7747/9/10/1270/s1, Figure S1: Life cycle of *C. bipunctata*, Figure S2: Scoring of gall formation, Figure S3: Effects of nutrient deficiency on seedling length, Figures S4–S6: Effects of plant hormone signaling on wallaby ear symptoms, Table S1: Culture media used to investigate effects of nutrient deficiency, Table S2: Statistical analyses.
