# Supplementary material for: Orange Leafhopper Cicadulina bipunctata Feeding Induces Gall Formation Nitrogen Dependently and Regulates Gibberellin Signaling
Source: Plants (Basel). 2020 Sep 26;9(10):1270. doi: 10.3390/plants9101270 (PMC7600891; doi:10.3390/plants9101270)
Supplement: Supplementary file 1 [file plants-09-01270-s001.pdf]

Table S1

Table S1 Culture media used to investigate effects of nutrient deficiency

Final concentration		Nutrient	Medium							
			C	–N	–P	–K	–S	–Mg	–Ca	–Fe
20.61	mM	NH_4NO_3	+	–	+	+	+	+	+	+
1.25	mM	KH_2PO_4	+	+	–	–	+	+	+	+
5.00	µM	KI	+	+	+	–	+	+	+	+
18.79	mM	KNO_3	+	–	+	–	+	+	+	+
0.10	mM	$MnSO_4$	+	+	+	+	–	+	+	+
29.91	µM	$ZnSO_4$	+	+	+	+	–	+	+	+
0.10	µM	$CuSO_4$	+	+	+	+	–	+	+	+
1.50	mM	$MgSO_4$	+	+	+	+	–	–	+	+
0.10	mM	$CaCl_2$	+	+	+	+	+	+	–	+
2.99	mM	$FeSO_4$	+	+	+	+	–	+	+	–
0.10	mM	H_3BO_3	+	+	+	+	+	+	+	+
0.11	µM	$CoCl_2$	+	+	+	+	+	+	+	+
0.10	mM	Na_2EDTA	+	+	+	+	+	+	+	+
1.03	µM	Na_2MoO_4	+	+	+	+	+	+	+	+
0.10	mM	$MnCl_2$	–	–	–	–	+	–	–	–
29.91	µM	$ZnCl_2$	–	–	–	–	+	–	–	–
0.10	µM	$CuCl_2$	–	–	–	–	+	–	–	–
1.50	mM	$MgCl_2$	–	–	–	–	+	–	–	–
2.99	mM	Fe-EDTA	–	–	–	–	+	–	–	–

C, control; +, supplied; −, absent.

Table S2

Table S2 Two-way ANOVA *p* values and *F* values

**Two-way ANOVA**		***F* value**	***p* value**
Plant height	Insect feeding	**4.08**	**<0.001**
	Media	**2.84**	**<0.001**
	Feeding × Media	**12.74**	**<0.001**
Plant height	Insect feeding	**3.99**	**<0.001**
	Nutrients	**2.16**	**<0.001**
	Feeding × Nutrients	2.16	0.07
Plant height	Insect feeding	**4.26**	**<0.001**
	PAC/GA	**3.40**	**<0.001**
	Feeding × Nutrients	**3.40**	**<0.001**

Figure S1

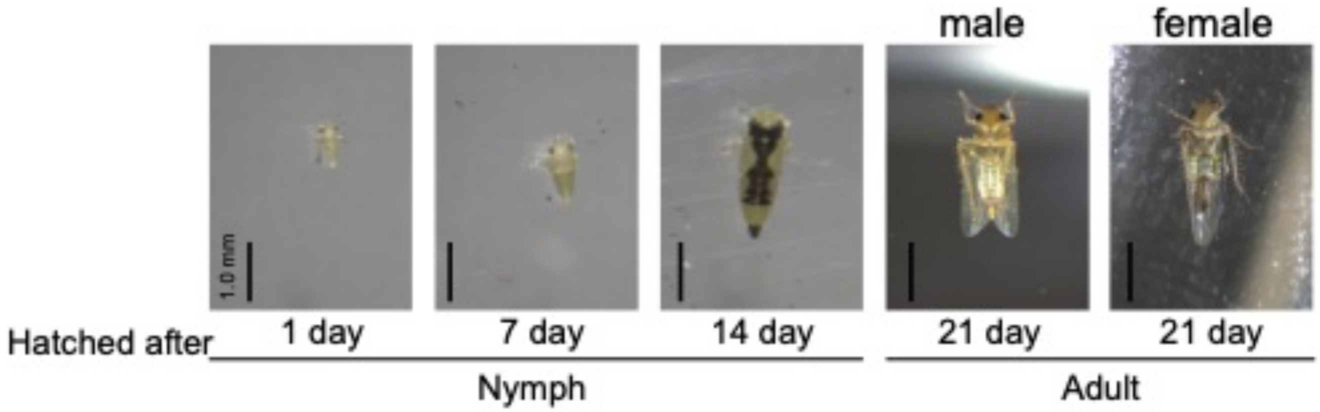

Figure S1. Life cycle of *C. bipunctata*. Hatched *C. bipunctata* nymphs become adults within three weeks.

Figure S2

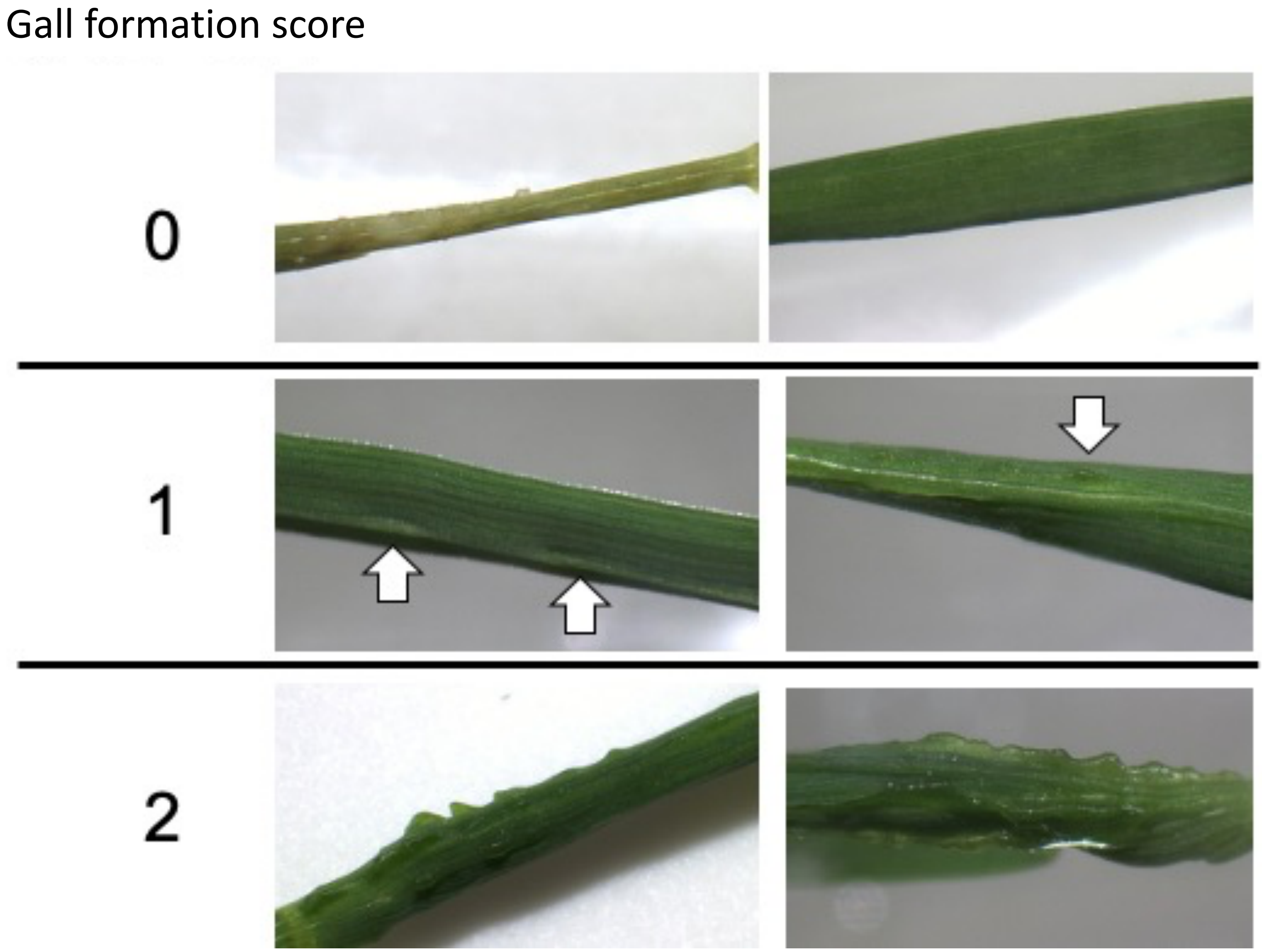

Figure S2. **Scoring of gall formation.** Symptom scores: 2 = tissues heavily swollen; 1 = veins partially thickened; and 0 = no visible symptoms. White arrows indicate the partially thickened-vein.

Miyazaki et al.,

Figure S3

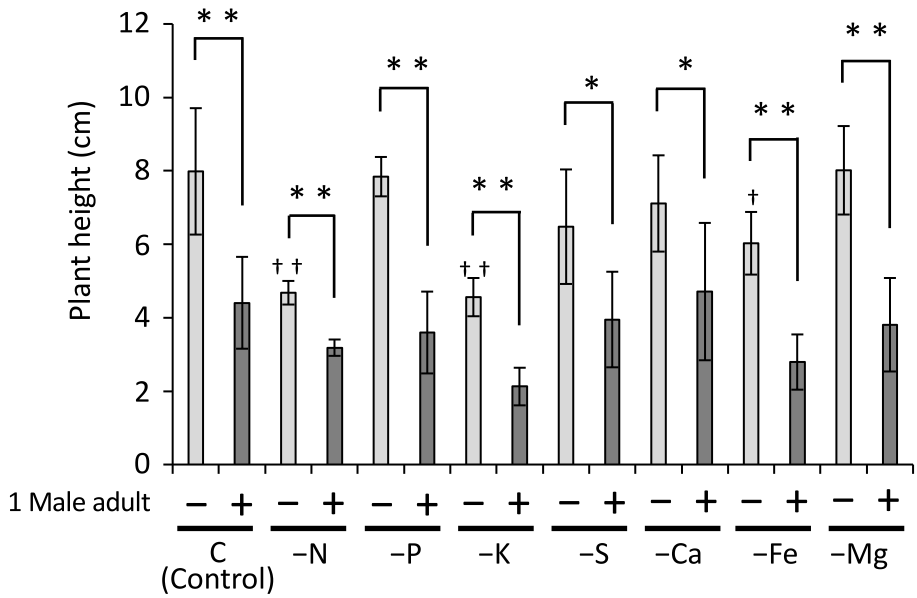

Figure S3. Effects of single nutrient deficiency on growth suppression. The rice seedlings were transferred onto new MS media without N, P, K, S, Ca, Mg, or Fe, and male *C. bipunctata* were added. Seedling length measured on day 4. The comparisons were made by two-way ANOVA (Table S2) with Dunnett's test († $p < 0.05$, †† $p < 0.01$ versus control) and *t* test (* $p < 0.05$, ** $p < 0.01$ versus non-feeding condition). Error bars indicate the standard deviation, $n = 5$.

Figure S4

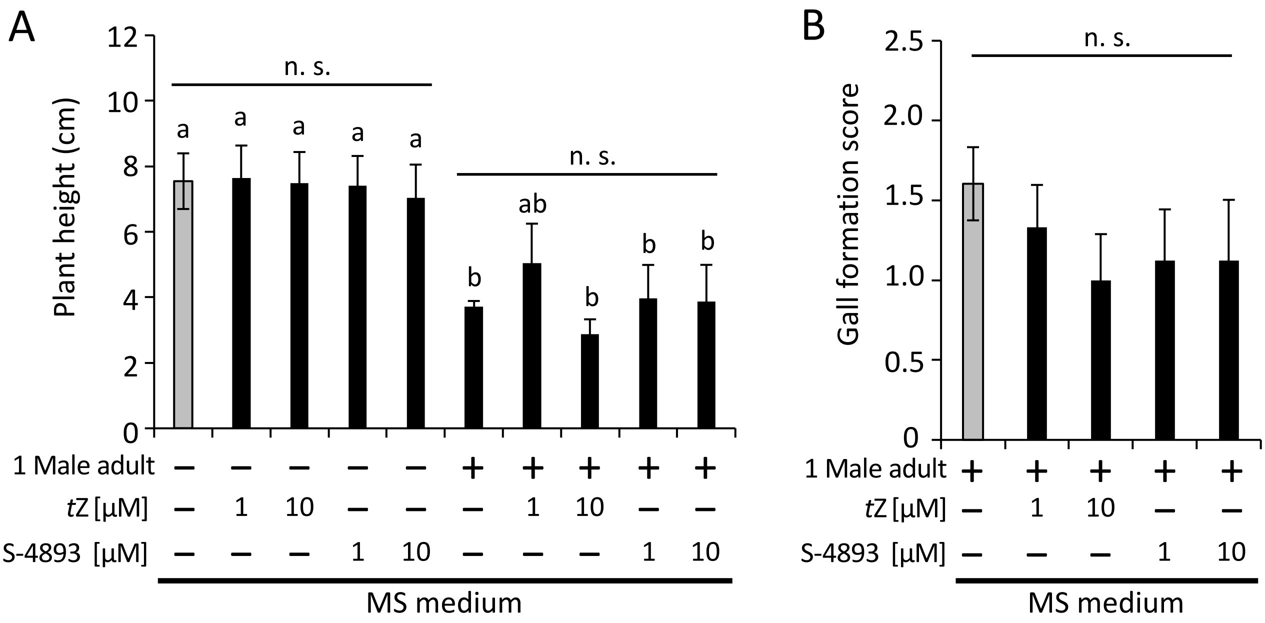

**Two-way ANOVA**		***F* value**	***P* value**
Plant height	Insect feeding	**4.03**	**<0.001**
	tZ/S4893	2.56	0.20
n = 6	Feeding × CK/S4893	2.56	0.52

Figure S4. Effects of cytokinin signaling on wallaby ear symptoms. Seedlings were placed on MS containing *tZ* and/or its inhibitor S-4893, or 0.1% DMSO as a mock control with or without a male of *C. bipunctata*. (A) Seedling length measured on day 4. The comparisons were made by two-way ANOVA and Tukey's HSD test. Bars with different letters indicate significant differences $p < 0.05$. Error bars indicate the standard deviation, $n = 6$. (B) Gall formation score recorded on day 10 after samples were transferred to each type of media. There is no significant interaction between insect feeding and *tZ*/S-4893 treatment (One-way ANOVA, $F(5, 24) = 2.62$, $n = 5$, $p = 0.43$). Error bars indicate the standard deviation, n. s.; not significant.

Figure S5

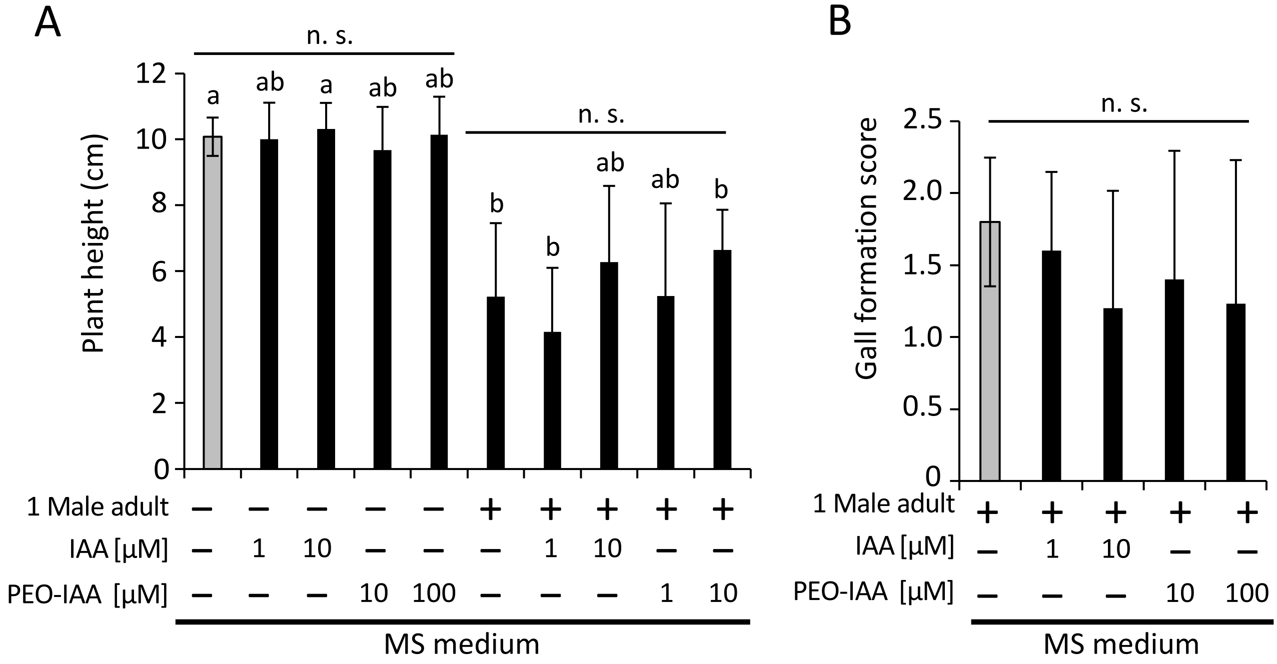

Two-way ANOVA		*F* value	*P* value
Plant height	Insect feeding	**4.03**	**<0.001**
	IAA/PEO-IAA	2.56	0.07
n = 6	Feeding × IAA/PEO-IAA	2.56	0.17

Figure S5. Effects of auxin signaling on wallaby ear symptoms. Seedlings were placed on MS containing IAA and/or its inhibitor PEO-IAA, or 0.1% DMSO as a mock control with or without a male of *C. bipunctata*. (A)) Seedling length measured on day 4. The comparisons were made by two-way ANOVA and Tukey's HSD test. Bars with different letters indicate significant differences $p < 0.05$. Error bars indicate the standard deviation, $n = 5$. (B) Gall formation score recorded on day 10 after samples were transferred to each type of media. There is no significant interaction between insect feeding and IAA/PEO-IAA treatment (One-way ANOVA, $F(4, 20) = 2.87$, $n = 5$, $p = 0.52$). Error bars indicate the standard deviation, n. s.; not significant.

Figure S6

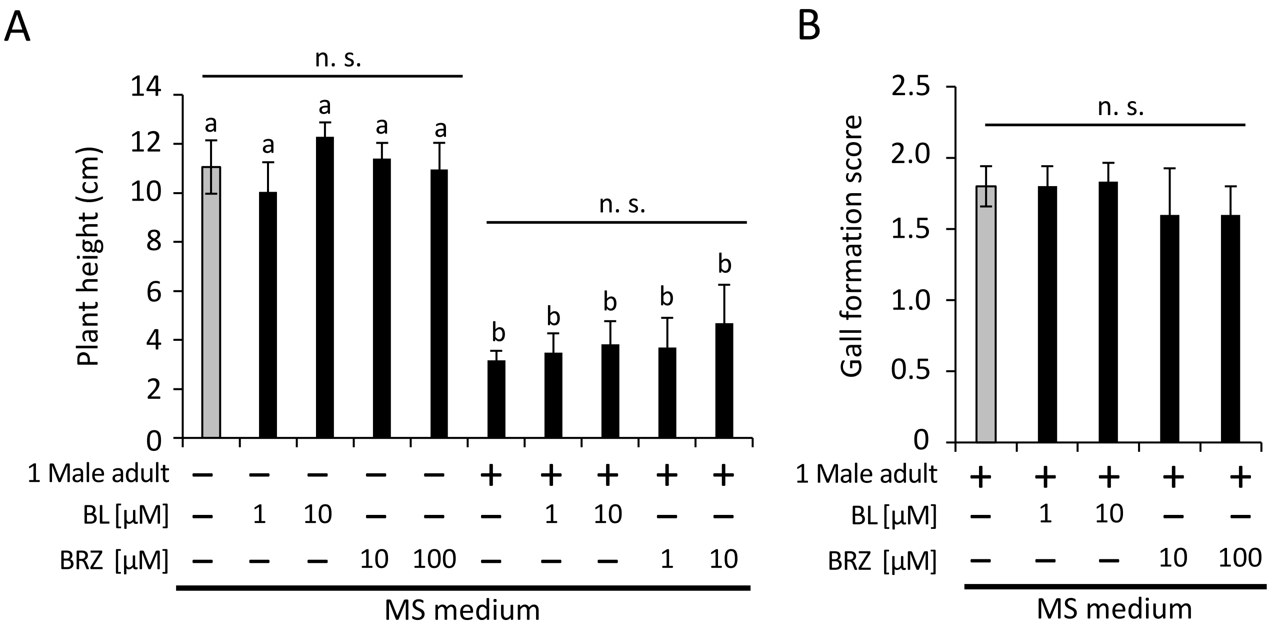

Two-way ANOVA		*F* value	*P* value
Plant height	Insect feeding	**4.08**	**<0.001**
	BL/Brz	2.61	0.09
n = 5	Feeding × BL/Brz	2.61	0.16

Figure S6. Effects of brassinosteroid signaling on wallaby ear symptoms. Seedlings were placed on MS containing BL and/or its inhibitor Brz, or 0.1% DMSO as a mock control with or without a male of *C. bipunctata*. (A)) Seedling length measured on day 4. The comparisons were made by two-way ANOVA and Tukey's HSD test. Bars with different letters indicate significant differences $p < 0.05$. Error bars indicate the standard deviation, $n = 5$. (B) Gall formation score recorded on day 10 after samples were transferred to each type of media. There is no significant interaction between insect feeding and BL/Brz treatment (One-way ANOVA, $F(5, 24) = 2.62$, $n = 5$, $p = 0.97$). Error bars indicate the standard deviation, n. s.; not significant.
